# Exploration of the factors influencing the quality of life among adolescents during the COVID-19 pandemic: the data from a cross-sectional study in Shandong

**DOI:** 10.1186/s12889-024-17981-5

**Published:** 2024-02-22

**Authors:** Hongjin Li, Zhiping Yang, Libin You, Shaojie Liu

**Affiliations:** 1 Institute for Infectious Disease Control and Prevention, Fujian Provincial Center for Disease Control and Prevention, 350012 Fuzhou, Fujian China; 2 Department of Clinical Nutrition, School of Medicine, The First Affiliated Hospital of Xiamen University, Xiamen University, 361003 Xiamen, China; 3 Key Laboratory of Fujian Province for Zoonotic Disease Research, Fujian Provincial Center for Disease Control and Prevention, 350012 Fuzhou, China

**Keywords:** COVID-19, Adolescence, Quality of life

## Abstract

**Background:**

The COVID-19 pandemic has sparked unprecedented transformations in the lives of adolescents, with reshaping their routines, social dynamics, educational experiences, and overall well-being. Our study delves into the influence of various factors on adolescents’ quality of life (QOL) among the COVID-19 pandemic in Shandong Province, China.

**Methods:**

Employing a cross-sectional research approach combined with multivariable analysis, we scrutinize the association of demographic factors (age, gender, education level, ethnic groups, urban area, and family economic status) and health-related behaviors (sleep duration, and self-reported health status) with QOL in 9953 students.

**Results:**

During the pandemic, the average QOL for adolescents in Shandong Province was 133. Our analysis revealed that sleep duration and age had statistically significant associations with total QOL, with the OR values of 1.43 (95% confidence interval (CI): 1.03 to 1.83) and 0.44 (95% CI: 0.19 to 0.70), respectively. Notably, we observed that adolescents from economically disadvantaged families, or those with poorer self-reported health status, were more likely to report lower QOL scores.

**Conclusions:**

Overall, our study highlights the potential association of sleep duration, age, family economic status, and self-reported health with the QOL of adolescents in Shandong Province during the pandemic. During similar public health crises, policymakers, educators, and healthcare providers can actively work through resource allocation and effective intervention measures towards alleviating financial burdens, improving health conditions, and ultimately enhancing the total QOL for adolescents.

## Introduction

As of 30 August 2023, more than 770 million confirmed cases of Coronavirus Disease 2019 (COVID-19) involving 6.95 million deaths across the world had been reported by the World Health Organization (WHO) (https://covid19.who.int/). The COVID-19 pandemic has resulted in sudden and unprecedented changes in the lives of adolescents around the world. Confronted with numerous fatalities and hundreds of thousands of individuals worldwide contracting the Severe Acute Respiratory Syndrome Coronavirus 2 (SARS-CoV-2), the majority of nations have implemented significant preventive measures. The previous research has indicated that the implementation of measures to mitigate the spread of COVID-19 has significant mental and physical health implications for students [1, 2]. The government may initiate proactive closure of schools to slow down the spread of disease during the delay phase, reduce the burden on healthcare systems, or safeguard at-risk populations during the mitigation phase. However, the protracted closure of schools may have serious and lasting social and health implications, particularly for economically disadvantaged children, possibly amplifying current disparities and exacerbating the effects of poverty [3, 4]. Quality of life (QOL) has become a crucial measure for individuals dealing with mental and physical health issues [5]. It assesses various dimensions of adolescents’ health, including physiological, psychological, emotional, and social aspects [6, 7]. Therefore, it is necessary to investigate the QOL among children and adolescents during the COVID-19 pandemic.

As the COVID-19 pandemic disrupted the daily routines and limited the social interactions, adolescents’ psychological well-being and overall QOL may also be significantly affected [8, 9]. Two studies conducted abroad utilized a cross-sectional research design to examine the current status of participants’ QOL during the COVID-19 pandemic [10, 11]. Existing research on COVID-19 indicated that adolescents’ QOL have deteriorated during the pandemic, with female adolescents reporting lower levels of QOL compared to male adolescents [12]. Adolescence is in a crucial and complex time with significant biological, psychological, and social changes and challenges, including the need for independence and social development [9, 13]. Some adverse health outcomes may be accompanied by a decline in QOL [14–16]. The QOL during adolescence may play a profound association with their adult health [17, 18]. Thus, understanding the situation and potential influencing factors of the QOL among children and adolescents during the unique phase of the COVID-19 pandemic is of great scientific significance.

Targeted intervention measures and support systems can address health issues caused by specific factors and alleviate the adverse association with the overall QOL for vulnerable populations during similar public health crises. In times of crisis, prioritizing resource allocation and implementing tailored intervention measures that account for the unique needs of different groups, while considering factors such as gender, age, socio-economic status, and other relevant determinants, is of importance [12, 19]. By implementing inclusive and equitable strategies, we can enhance the well-being and promote equal opportunities for individuals across society, thereby mitigating the adverse effects of the crisis. To our knowledge, there have been few studies investigating the QOL among adolescents in China. Our aim is to use a cross-sectional analysis to describe the QOL scores of adolescents in Shandong, China, and potential contributing factors for the scores.

## Materials and methods

### Participants and study design

The prior multi-wave survey was conducted in Shandong Province, China, across academic years 2015/2016, 2016/2017, 2017/2018, and 2020/2021 [20]. It received institutional ethical approval and included 17 cities. A combination of random and Probabilistic Proportionate Size (PPS) sampling methods was used to select 100 schools from 10 administrative districts in the 2020/2021 academic year, ensuring representation across specific geographic, population, and socio-economic factors and excluding those with fewer than 100 students in each grade or less than 300 students in total [20]. A total of 99,327 students from 186 secondary schools in 17 cities of Shandong Province participated in the survey. A detailed protocol of study design had been published by Shengfa Zhang et al. [20].

**Fig. 1:**
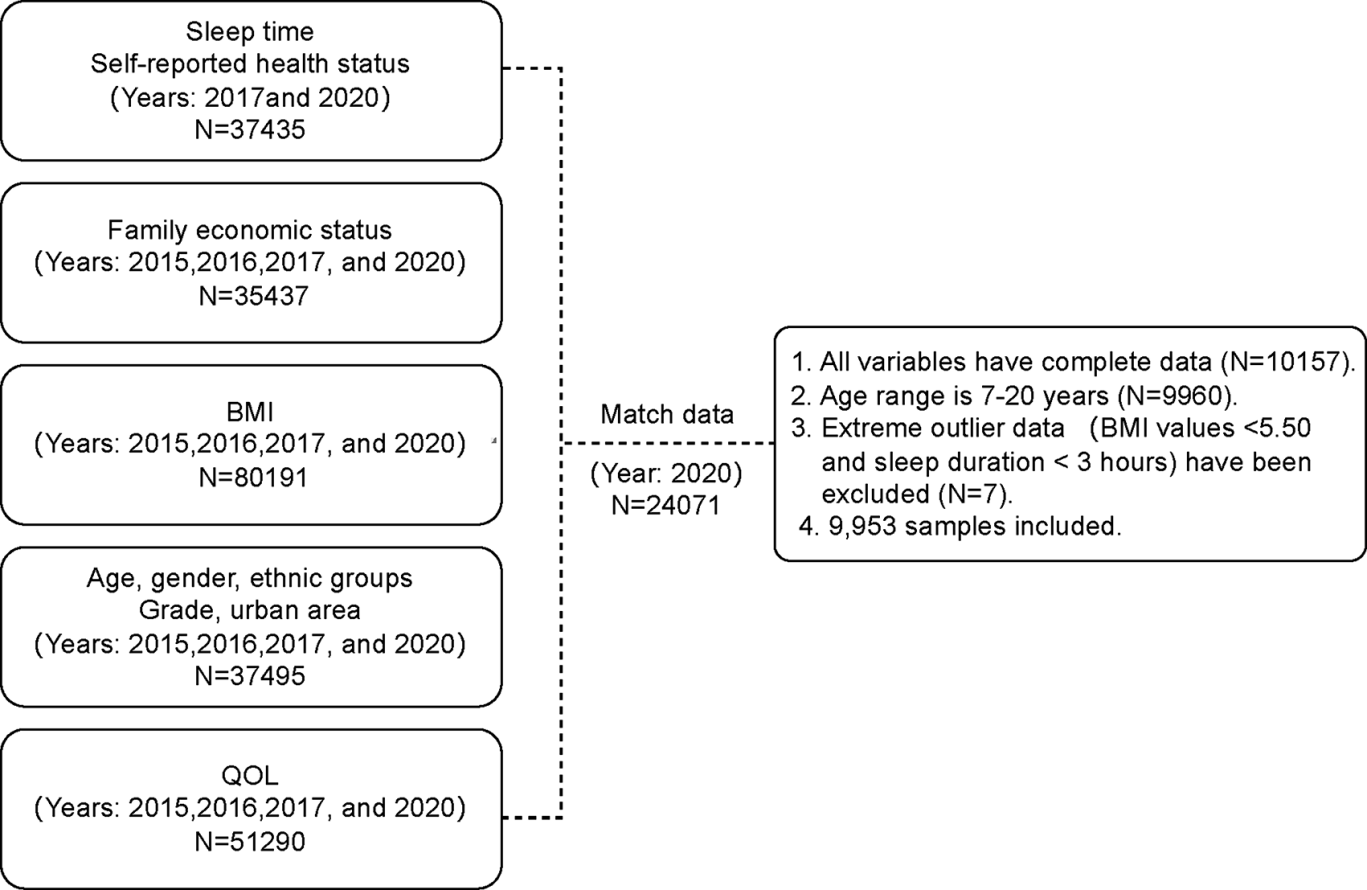
The process flowchart of participant screening in our study population

Based on previous research databases [20], we conducted a cross-sectional study using survey data from 2020 to 2021 to examine the factors influencing the QOL of adolescents in Shandong Province during the COVID-19 pandemic. As shown in the flowchart (Fig. 1), the variables in this study were obtained from five questionnaires. The variables from top-left to bottom-left were sourced from five different datasets, namely: nutrition and diet, social-economic status, physical fitness test, individual information, and QOL. Based on the students’ ID numbers, we integrated all variables included in this study in 2020 into a large dataset with a sample size of 24,071. Then, we filtered out the complete dataset (N = 10,157), including individuals aged 7–20 years, and excluded outliers in BMI and sleep duration. Due to incomplete demographic information, a total of 7 students were excluded from our study. Finally, 9953 individuals met our exclusion and inclusion criteria with completing this study. This study has been approved by the Ethics Committee of Shandong University (Grant Number: 20180517) [7].

### The quality-of-life scale for children and adolescents (QLSCA)

To evaluate the QOL of middle school students in Shandong Province, China, we utilized the assessment tool of the QLSCA. The QLSCA is a Chinese version of the QOL Scale for Children and Adolescents, consisting of 49 items for measuring 13 dimensions of students’ lives, such as their relationships with teachers and parents, partnership with fellow students, learning abilities and attitudes, self-perception, physical well-being, negative emotions, attitudes towards homework, living environment convenience, social activities, sports capacity, self-satisfaction, and other unspecified factors [20]. The QLSCA uses a five-point Likert-type scale to measure either frequency or intensity, with a recall period of two weeks. Scores were calculated for each dimension, with higher scores indicating a better QOL. The Chinese version of the QLSCA has been shown to have acceptable reliability and validity in previous studies [7]. In this study, the Cronbach’s alpha coefficient for the QOL scale we employed was calculated to be 0.885 [21].

### Questionnaire survey and physical fitness tests

The questionnaire survey was conducted by trained investigators. Trained surveyors were recruited from physical education teachers to conduct questionnaire surveys and physical fitness tests in each school using standardized procedures, as previously reported by the researchers [20]. Data collection was aligned with the annual physical fitness test mandated by the Ministry of Education in China, utilizing online questionnaires surveys [20]. The questionnaires used in this study had a response rate of over 36%. Demographic characteristics (age, gender, education level, ethnic groups, urban area, and family economic status) and health-related behaviors (sleep duration, and self-reported health status) were obtained from the questionnaire surveys, while height and weight measurements were collected during the physical fitness tests. Height and weight were recorded, with accuracies within 0.1 cm and 0.1 kg, respectively. The physical examination data were acquired based on the students’ most recent medical examination reports. After ensuring personal privacy through anonymization, data processing was performed using Python 3.7 by two graduate students under the guidance of experts in adolescent health and the supervision of two staff members specialized in scientific data. The detailed information has been discussed in the relevant published literature [20]. Please refer to the literature for more information.

### Primary variables and covariables

Continuous variables were described as mean (standard deviation, SD), including sleep duration, age, BMI, and QOL; categorical variables were described as frequency (ratio), including sleep duration, age, gender, ethnic groups, education level, family economic status, self-reported health status, and urban area. Sleep duration was defined as the duration of nighttime sleep and does not include nap time. Ethnic group was sort as Han and Ethnic minorities, including Mongolian, Manchu, Hui, Zang, and Others. Family economic status: Lowest: The lowest income or socioeconomic class, Lower-Middle: Lower-middle income or socioeconomic class, Middle: Middle income or socioeconomic class, Upper-Middle: Upper-middle income or socioeconomic class, Highest: The highest income or socioeconomic class. The self-reported health status is divided into five categories: Excellent, very good, good, fair, and poor. We chose the median age (14 years old) as the criterion for grouping, resulting in two sub-groups: 7–14 years old and above 14 years old. According to previous research [22], we selected the sleep duration (9 h) as the criterion for grouping, resulting in two sub-groups: below 9 h (sleep deprivation) and 9 h and above (adequate sleep). Gender was classified as male and female. Body Mass Index (BMI, kg/m^2^) was calculated according to the formula BMI (kg/m^2^) = weight (kg)/height^2^ (m^2^). In our study, we surveyed students from both middle school and high school grades (junior or senior high school), which aligns with the school categorization used in previous research [20].

### Statistical analyses

Statistical analyses were done through employing SPSS version 26 (IBM, Chicago, IL, USA). Descriptive statistics were calculated for all characteristics in term of sleep duration, age, gender, BMI, education level, family economic status, self-reported health status, and QOL. Student-t test was used for comparing the difference of total QOL among these variables that sleep duration, age, gender, and education level. We applied one-way ANOVA to investigate the differences in health status based on ethnicity, region, family economic status, and self-reported health status. Based on the linear regression method, we used R software version 4.2.2 for both univariable analysis and multivariable analysis. Multivariable analysis was performed to assess the association among sleep duration, age, gender, BMI, education level, family economic status, self-reported health status, ethnic groups, urban area and QOL and to identify β and the corresponding 95% confidence intervals (CI). Two-sided *P* values < 0.05 were considered statistically significant.

## Results

As shown in Table 1, the majority of surveyed students (75.41%) indicated that they had achieved a sufficient amount of sleep. The majority of surveyed students were aged 7–14 (63.81%), and the ratio of male to female ratio was closer to 1:1. The majority of the surveyed participants, accounting for 68%, consisted of middle school students. The highest proportion, accounting for 76.70%, was represented by students with a moderate family financial situation. The highest proportion of surveyed students, accounting for 40.32%, self-reported their health condition as “very good”. The proportion of Han Chinese students among the survey participants was 99.25%. The average sleep duration of the surveyed students was 8 h. The average age of the surveyed students was 14 years old. The average BMI of the surveyed students was 20.57 kg/m^2^. The average QOL score for the surveyed students was 133.

**Fig. 2:**
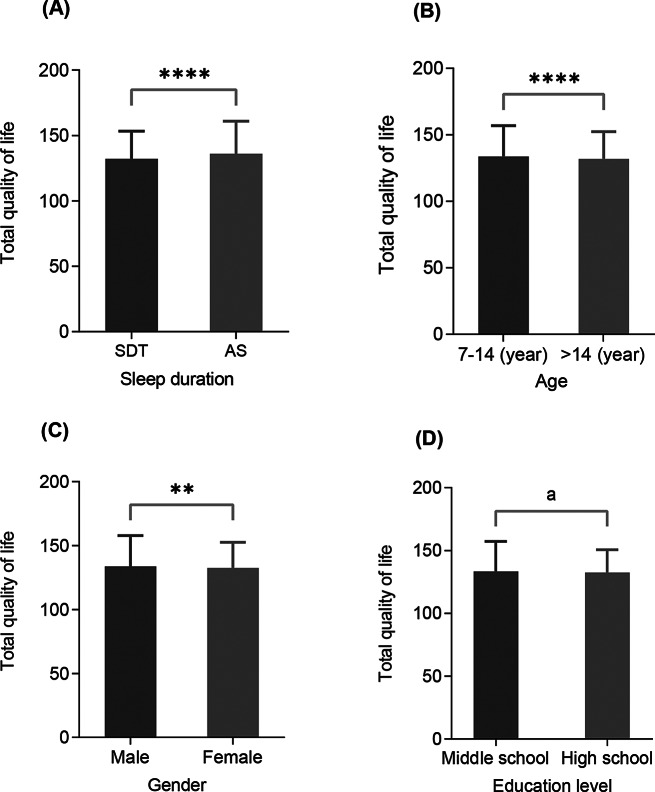
Differences in total quality of life among sleep duration, age, gender, and education level. (**A**), sleep duration, below 9 h (sleep deprivation, SDT) and 9 h and above (adequate sleep, AS); (**B**), age; (**C**), gender; (**D**), education level. **, p-value is less than 0.01; ****, p-value is less than 0.0001. “a” indicates that there is no statistically significant difference in the results

As illustrated in Fig. 2, we found that sleep, gender, and age can all have a significant association with overall QOL. Our research findings indicated that study participants who sleep more than 8 h per day have a higher QOL than those who sleep for 8 h or less. Gender differences were found in QOL, with males reporting higher total QOL than females. Age was also a factor affecting total QOL for adolescents. The participants aged 7–14 exhibited a higher QOL compared to those aged 14 and above. We did not find significant differences in the total QOL between middle school and high school.

**Table 1:** Characteristics of study participants

Characteristics	Categories	n (%) or mean ± SD
**Sleep duration (h), n (%)**	Sleep deprivation	7506 (75.41)
	Adequate sleep	2447 (24.59)
**Age (years), n (%)**	7–14	6329 (63.81)
	> 14	3589 (36.19)
**Gender, n (%)**	Male	4966 (49.89)
	Female	4987 (50.11)
**Education level, n (%)**	Middle school	6838 (68.70)
	High school	3115 (31.30)
**Family economic status, n (%)**	Lowest	360 (3.62)
	Lower-Middle	1195 (12.01)
	Middle	7634 (76.70)
	Upper-Middle	589 (5.92)
	Highest	175 (1.76)
**Self-reported health status, n (%)**	Excellent	2393 (24.04)
	Very good	4013 (40.32)
	Good	2100 (21.1)
	Fair	1304 (13.1)
	Poor	143 (1.44)
**Ethnic groups, n (%)**	Han	9878 (99.25)
	Ethnic minorities	75 (0.75)
**Urban area, n (%)**	Ji’nan	457 (4.59)
	Dongying	1201 (12.07)
	Weifang	717 (7.20)
	Jining	1578 (15.85)
	Weihai	1049 (10.54)
	Dezhou	1547 (15.54)
	Liaocheng	925 (9.29)
	Linyi	1094 (10.99)
	Heze	1385 (13.92)
**Sleep duration (h), mean ± SD**		8 ± 1
**Age (years), mean ± SD**		14 ± 2
**BMI (kg/m** ^**2**^ **), mean ± SD**		20.57 ± 4.19
**Total quality of life, mean ± SD**		133 ± 22

*Abbreviation* BMI, body mass index; SD, standard deviation. Family economic status: Lowest: The lowest income or socioeconomic class, Lower-Middle: Lower-middle income or socioeconomic class, Middle: Middle income or socioeconomic class, Upper-Middle: Upper-middle income or socioeconomic class, Highest: The highest income or socioeconomic class

**Fig. 3:**
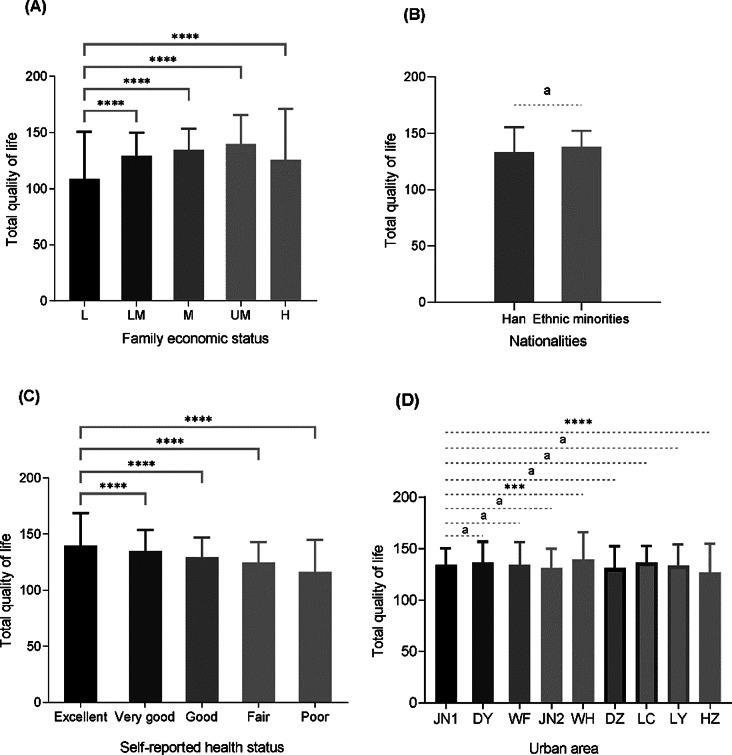
The differences in total quality of life among economic condition, nationalities, self-reported health status, and urban area. (**A**), family economic status: L (Lowest): The lowest income or socioeconomic class, LM (Lower-Middle): Lower-middle income or socioeconomic class, M (Middle): Middle income or socioeconomic class, UM (Upper-Middle): Upper-middle income or socioeconomic class, H (Highest): The highest income or socioeconomic class; (**B**), ethnic groups, Han and Ethnic minorities; (**C**), self-reported health status; (**D**), urban area, JN1, DY, WF, JN2, WH, DZ, LC, LY, and HZ represent the cities of Ji’nan, Dongying, Weifang, Jining, Weihai, Dezhou, Liaocheng, Linyi, Heze, respectively. ***, p-value is less than 0.001; ****, p-value is less than 0.0001. “a” indicates that there is no statistically significant difference in the results

As shown in Fig. 3 (A), we found that lower levels of socioeconomic status were associated with lower total QOL. Figure 3 (B) compared the total QOL among different groups stratified by ethnicity. We did not find any difference in the QOL between Han students and the compared groups of Ethnic minorities. Figure 3 (C) shown that a heightened level of self-reported health status corresponds to an enhanced total QOL. In Fig. 3(D), the total QOL was compared among different groups stratified by urban area. We found that the total QOL among students in Jinan is significantly higher than in Heze but lower than in Weihai.

As shown in Table 2, both univariable and multivariate regression analyses have demonstrated the significant association of sleep duration, age, family economic status, and self-reported health status with total QOL during the COVID-19 pandemic. Specifically, it observed that adolescents with lower economic conditions tended to exhibit poorer total QOL, while those with better self-reported health status displayed better total QOL. Additionally, we observed the significant associations between sleep duration and age with total QOL. In the multivariate regression analyses, the effect estimates for sleep duration and age associated with total QOL were 1.43 (with a 95% CI of 1.03 to 1.83) and 0.44 (with a 95% CI of 0.19 to 0.70). Importantly, the results obtained from multivariate regression analyses supported the findings of the univariable regression analysis in terms of economic condition, self-reported health status, and sleep duration (Table 2). In addition, we consistently found in both univariable analysis and multivariable analysis that the total QOL among students in Jinan was significantly higher than that of students in Jining, Dezhou, and Heze.

**Table 2:** The β (95%CI) of factors to total quality of life from univariable analysis and multivariable analysis

Variable	N	Univariable analysis		Multivariable analysis	
		β (95% CI)	P value	β (95% CI)	P value
**Sleep duration (h)**	9953	2.61 (2.24, 2.98)	< 0.001	1.43 (1.03, 1.83)	< 0.001
**Age (year)**	9953	-0.51 (-0.75, -0.28)	< 0.001	0.44 (0.19, 0.70)	< 0.001
**Gender**					
Male	4966	Reference		Reference	
Female	4987	-1.21 (-2.08, -0.34)	0.01	-0.45 (-1.28, 0.38)	0.29
**BMI**	9953	0.12 (0.01, 0.22)	0.03	0.07 (-0.03, 0.17)	0.17
**Family economic status**					
Lowest	360	Reference		Reference	
Lower-Middle	1195	20.82 (18.29, 23.35)	< 0.001	21.77 (19.33, 24.21)	< 0.001
Middle	7634	26.08 (23.80, 28.35)	< 0.001	25.50 (23.31, 27.70)	< 0.001
Upper-Middle	589	31.08 (28.26, 33.90)	< 0.001	28.27 (25.55, 30.99)	< 0.001
Highest	175	17.21 (13.32, 21.09)	< 0.001	15.37 (11.63, 19.10)	< 0.001
**Self-reported health status**					
Excellent	2393	Reference		Reference	
Very good	4013	-5.07 (-6.16, -3.99)	< 0.001	-5.53 (-6.60, -4.47)	< 0.001
Good	2100	-10.79 (-12.05, -9.53)	< 0.001	-10.40 (-11.65, -9.15)	< 0.001
Fair	1304	-15.14 (-16.59, -13.69)	< 0.001	-13.68 (-15.13, -12.23)	< 0.001
Poor	143	-23.54 (-27.17, -19.91)	< 0.001	-18.77 (-22.29, -15.25)	< 0.001
**Ethnic groups**					
Han	9878	Reference		Reference	
Ethnic minorities	75	4.87 (-0.16, 9.90)	0.06	3.91 (-0.78, 8.61)	0.10
**Urban area**					
Ji’nan	457	Reference		Reference	
Dongying	1201	2.52 (0.17, 4.88)	0.04	1.96 (-0.29, 4.21)	0.09
Weifang	717	0.33 (-2.23, 2.90)	0.799	-0.92 (-3.35, 1.51)	0.46
Jining	1578	-3.05 (-5.33, -0.78)	0.01	-2.30 (-4.45, -0.15)	0.04
Weihai	1049	5.22 (2.82, 7.62)	< 0.001	1.79 (-0.53, 4.11)	0.13
Dezhou	1547	-3.10 (-5.38, -0.82)	< 0.01	-3.22 (-5.38, -1.05)	< 0.01
Liaocheng	925	2.23 (-0.22, 4.68)	0.07	1.41 (-0.92, 3.75)	0.24
Linyi	1094	-0.64 (-3.03, 1.74)	0.60	-1.85 (-4.11, 0.41)	0.11
Heze	1385	-7.30 (-9.61, -4.99)	< 0.001	-6.41 (-8.61, -4.21)	< 0.001

Family economic status, Lowest: The lowest income or socioeconomic class, Lower-Middle: Lower-middle income or socioeconomic class, Middle: Middle income or socioeconomic class, Upper-Middle: Upper-middle income or socioeconomic class, Highest: The highest income or socioeconomic class

## Discussion

The COVID-19 pandemic has a significant association with the lives of adolescents worldwide. This study aims to explore the factors affecting the total QOL in adolescents during the COVID-19 epidemic in Shandong Province, China. Our study found that during the COVID-19 outbreak in Shandong Province, there was a significant association between demographic factors and QOL among adolescents. Specifically, adolescents with lower household income and younger adolescents were found to have lower QOL scores. The results of this study may provide a certain of reference for policy development, as they can help identify areas where need to provide the support and assistance for alleviating stress experienced by adolescents during the pandemic. Additionally, this research may contribute to increasing public awareness and understanding of adolescent health issues.

The findings of our study highlight the importance of providing targeted assistance to economically disadvantaged students, who may experience a lower QOL compared to their counterparts during the COVID-19 lockdown measures, in line with previous research findings [23, 24]. Several studies have found that economic status had an important influence for the QOL during childhood [25]. During the early stages of life, there is a connection between economic disadvantage and unfavorable dietary habits, disrupted sleep patterns, and later adoption of unhealthy behavioral lifestyles such as smoking and alcohol consumption [26–28]. Moreover, economic disadvantage during early life is associated with an increased risk of various health issues in later life, including cardiovascular metabolic risk factors, diabetes, fatty liver disease, impaired lung function, and depression [29–33]. Additionally, during adolescence, economic disadvantage is linked to a higher risk of developing metabolic syndrome [34].

Economic status may have a profound association with adolescent health, and given the COVID-19 pandemic’s unique circumstances, this association may be further accentuated due to potential disparities that arise [35–38]. School closures during the pandemic may exacerbate the educational achievement gap between students from low-income and high-income families, as those from low-income backgrounds may lack access to essential resources for at-home online learning [3]. Furthermore, the economic downturn triggered by the pandemic could intensify child poverty, which may have enduring impacts on their health and well-being [3]. During the ongoing COVID-19 pandemic, there is an urgent need to address the impact of socioeconomic disparities that may exacerbate the effects of the public health crisis. By focusing on these high-risk factors, public health interventions can be better tailored to meet the unique challenges and needs of vulnerable children and promote health equity for all. Therefore, it is crucial to explore these potential disparities and their effects on adolescent health during these uncertain times, to ensure that all individuals have equal access to education and healthcare resources.

Our research results indicate that sleep duration may be a potential factor associated with QOL among adolescents during the pandemic. According to previous research findings, an ample allocation of sleep has been associated with a favorable influence on health-related QOL [39]. In the current investigation, we unveil those individuals who obtained sufficient sleep during the COVID-19 pandemic reported higher QOL. These results underscore the importance of acquiring an adequate amount of sleep for overall well-being, especially in the presence of stressors and uncertainties. Previous studies have indicated that long-term mobile phone use and excessive screen time can have an association with the sleep, psychological health, and QOL of adolescents [7, 40, 41]. High levels of physical activity have been found to mitigate the negative effects of excessive screen time [42]. Moreover, increasing physical activity is considered more crucial for improving the health of adolescents than simply reducing screen time [43]. In addition, our findings indicate that the QOL among adolescents in Jinan was significantly higher compared to their counterparts in Jining, Dezhou, and Heze. Previous research suggests that the roles of transport and land use, urban nature, public space, facilities and services, housing, and information and communications technology in the QOL within cities underwent a transformation during COVID-19 [44]. The influence of these urban factors on adolescent health appears to be complex and requires further study to be properly understood.

Our findings may provide a certain of reference for policymakers and healthcare professionals in developing interventions to improve the QOL of adolescents during the COVID-19 outbreak. For instance, targeting interventions towards these specific demographic groups may be effective in reducing disparities in QOL and promoting overall well-being. Additionally, addressing the unique needs and challenges faced by these groups may be important in designing effective interventions. In an ideal scenario, interventions such as exercise, collaboration with mental health professionals, academic planning and support, and parent-adolescent communication interventions have the potential to significantly enhance QOL and subsequently improve physical and/or mental health [45–49]. These comprehensive measures not only contribute to the physical well-being of adolescents but also foster the development of their mental health, establishing a strong foundation for their growth and well-being.

The study has some limitations that need to be considered to ensure the validity of its findings. In multivariable regression analysis, the absence of a significant association of gender differences with adolescents’ QOL during the COVID-19 pandemic, contrary to previous findings [8, 12, 19], could be attributed to various factors such as sample size and regional specificity. Replicating the study in multiple regions or countries can help establish generalizability. Additionally, the cross-sectional design of the study also limited the ability to establish cause-and-effect relationships between the demographic characteristics and adolescents’ QOL during COVID-19 pandemic. Future research should explore more longitudinal designs to better understand their relationship. Furthermore, due to the scarcity of data on COVID-19 infections among adolescents, we are unable to accurately assess the potential confounding effect of novel coronavirus infection on our study findings. Lastly, as mentioned previously, the study had limitations in terms of the factors considered. Including additional factors like lifestyle behaviors, dietary habits, and other variables would enhance the understanding of the impact of the pandemic on adolescent health. Future research should aim to address these limitations to further advance the knowledge in this area.

## Conclusion

In conclusion, our study reveals the adverse effects of sleep deprivation, low socio-economic status, and self-reported poor health on the QOL of adolescents during a specific period, emphasizing the importance of addressing these problems for improving their happiness. These findings are of significant importance in addressing the economic and health challenges faced by adolescents and enhancing their overall QOL. By allocating resources and implementing effective interventions, policymakers, educators, and healthcare providers can collaborate actively to alleviate the financial burden, improve health outcomes, and ultimately enhance the QOL of adolescents.

## Data Availability

After obtaining approval from the National Population Health Data Center, the data (https://www.ncmi.cn//phda/dataDetails.do?id=CSTR:17970.11.A0031.202107.209.V1.0) will be available for use.
